# Anti-sulfatide antibody associated GBS with headache and abdominal pain: a case report

**DOI:** 10.1186/s12887-023-04287-5

**Published:** 2024-06-26

**Authors:** Jia Zhang, Jing Gan, Jianjun Wang

**Affiliations:** 1 Department of Pediatrics, West China Second University Hospital, Sichuan University, Chengdu, 610041 China; 2 Key Laboratory of Obstetrics & Gynecologic and Pediatric Diseases and Birth Defects of the Ministry of Education, Sichuan University, Chengdu, Sichuan China

**Keywords:** Anti-sulfatide antibodies, GBS, Dilated ventricles, Urinary calculus

## Abstract

**Background:**

Guillain‒Barre syndrome (GBS) is an acute inflammatory peripheral neuropathy caused by autoimmunity. Gangliosides and sulfatides are important components of peripheral nerves. Anti-sulfatide antibody-mediated complement is associated with acute sensorimotor peripheral neuropathy in GBS, which is characterized by pain and paresthesias.

**Case presentation:**

The child was a 7-year-old girl with headache and abdominal pain, followed by limb numbness and pain. Cranial imaging showed ventricular dilatation, peripheral nerve function conduction examination showed polyradiculopathy, and cerebrospinal fluid tests showed normal cell counts but elevated protein levels, all of which led to the diagnosis of GBS. After treatment with intravenous immunoglobulin (400 mg/kg × 5 days), the symptoms did not improve, and muscle strength progressively worsened, accompanied by paroxysmal complexion flushing, heart rate fluctuation, hyperhidrosis, and a progressive increase in cerebrospinal fluid protein (up to 3780.1 mg/L). On the basis of these findings combined with serum anti-sulfatide IgM positivity, anti-sulfatide antibody-related GBS was considered, and treatment with low-dose prednisolone (1 mg/kg/d) led to symptom improvement.

**Conclusions:**

Anti-sulfatide antibody-associated GBS is associated with small fiber peripheral neuropathy. The main manifestations are pain, paresthesias and autonomic dysfunction. In addition to the dysfunction of spinal nerve root absorption caused by increased cerebrospinal fluid protein, autonomic dysfunction may be involved in pain. When the therapeutic effect of immunoglobulin is not satisfactory, a low dose and short course of corticosteroids can be considered, and the prognosis is good.

## Background

Guillain‒Barre syndrome (GBS) spectrum disorders in children are a group of autoimmune diseases that damage the central and/or peripheral nervous system. The etiology and pathogenesis of GBS are not completely clear but may be related to infection and immune abnormalities. Anti-perineurial surface glycolipid antibodies are closely related to the pathogenesis of GBS spectrum diseases, including anti-ganglioside antibodies and anti-sulfatide antibodies, which are important biomarkers for the diagnosis of GBS spectrum diseases. This case report summarizes the clinical data of a child with anti-sulfatide antibody-related GBS diagnosed and treated in West China Second Hospital of Sichuan University, as well as the results of a literature review, to improve the diagnosis and treatment of this disease.

## Case presentation

The child, a girl aged 7 years and 9 months, was admitted to the hospital with "headache and abdominal pain for 12 days and numbness and pain in the limbs for 4 days", during which she had cold symptoms such as low fever and itching all over the body, without rash. She was treated with cetirizine orally. The pruritus slightly improved, but she still had a headache, abdominal pain, limb numbness, and pain in both lower limbs. Brain CT showed dilatation of the temporal horn, lateral ventricle, third ventricle, and fourth ventricle. Abdominal ultrasound showed no abnormalities. Then, the patient was admitted to our hospital for further treatment. The child had a milk protein allergy. More than 1 year before admission, the child was found to have a cyst in the suprasellar cisterna during a physical examination. Head CT showed no abnormality after an operation for the cyst. The child was born G2P1 and 36 weeks premature, with a birth weight of 2700 g. At admission, the child’s weight was 21 kg (P10~P25), height was 118 cm (P3~P10), and psychomotor development was normal. No abnormalities were noted in the family history. Physical examination findings at admission were as follows: blood pressure was 98/61 mmHg, abdominal was normal, and tenderness was noted in the groin and lower limbs. Limbs numbness, normal depth sensation, normal muscle strength, and muscle tension were also noted. Deep tendon reflexes were absent, and pathological signs, meningeal irritation, ataxia negativity, and a positive straight leg elevation test were observed.

**Fig. 1:**
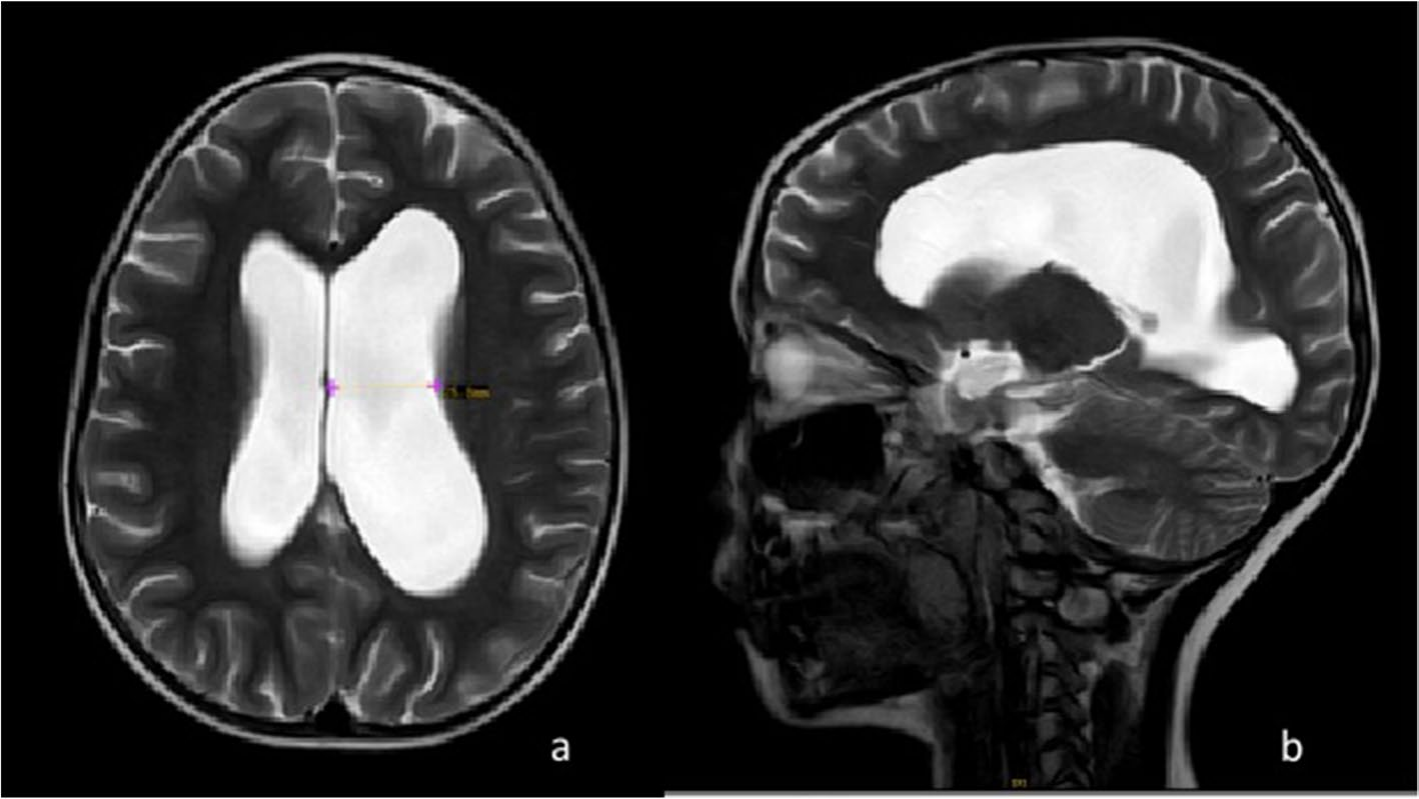
Brain MRI (T2WI) showed dilated ventricles, especially the left lateral ventricle

After admission, relevant auxiliary examinations were performed, and the results were as follows: routine blood, biochemical, autoimmune, and etiological screening results were all normal. Anti-Streptolysin O (ASO) was 2320 IU/ml, and group A streptococcus nucleic acid test results were negative. The cell count in cerebrospinal fluid (CSF) was 0 × 10^9/L, protein was 1687.6 mg/L, glucose was 4.4 mmol/L, and test results for pathogens were negative. The CSF IgG index was 0.69. The 24-h synthesis rate of IgG was 57.35 mg. The results for tests for antibodies against gangliosides (GQ1b/GD1b/GM3/GM1), autoimmune encephalitis-associated antibodies (NMDAR/AMPA1/AMPA2/GAGBB/LGI1/CASPR2/GAD65/MOG) and oligoclonal bands (OB) were negative. Ultrasound of the liver, bile, pancreas, spleen, urinary system, and gastrointestinal tract and EEG showed no abnormalities. An electrophysiological study demonstrated severe multifocal demyelination, which was evident in the lower limbs. SNAP was elicited and poorly differentiated, and SCV was reduced. Somatosensory evoked potentials demonstrated prolonged latency. Brain MRI showed a widening of the prepontine cisterna and suprasellar cisterna and the dilatation of the fourth and third ventricles and bilateral lateral ventricles, especially the left lateral ventricle (Fig. 1). According to the clinical characteristics and laboratory and electrophysiological examination results, the child was diagnosed with GBS. After treatment with intravenous immunoglobulin (400 mg/kg × 5 days), nerve growth factor and vitamin B supplements, the child's symptoms were not relieved. The pain was not relieved, and the muscle weakness was worse than before. The muscle strength of both upper limbs was grade 4, and that of the lower limbs was grade 3, especially in the proximal region. A high dose of immunoglobulin (1 g/kg) was given again, and mannitol was given to reduce intracranial pressure. CSF showed a progressive increase in protein of 3411.8 mg/L, an IgG index of 1.18, and an IgG 24-h synthesis rate of 82.03 mg; cytology and glucose were normal. The test result for serum anti-sulfatide IgM was positive. MRI of the whole spinal cord showed no definitive abnormality.

The child repeatedly complained of headache, abdominal pain and limb numbness, and the pain was not relieved. The degree of pain was relatively severe, and pain was accompanied by paroxysmal flushing, heart rate fluctuation, and hyperhidrosis. After antipyretic analgesics and antispasmodic drugs, pain could sometimes be relieved, but it would return. Multiple CSF reexaminations showed elevated protein (up to 3780.1 mg/L) and CSF pressure up to 170 mmH2O, as well as serum anti-sulfatide IgM positivity. Considering the positivity for anti-sulfatide antibody associated with GBS, prednisolone (1 mg/kg, gradually reduced after 1 week) was added. After 2 weeks of treatment, the muscle strength of the limbs recovered, and no pain was reported. During this period, the child had a fever once and then transient gross hematuria accompanied by elevated blood pressure (101-142/55-106 mmHg). Repeated urine examination suggested nonglomerular hematuria, an erythrocyte sedimentation rate (ESR) of 64 mm/h, ANA positivity (1:100), and an ASO of 1330 IU/mL. Urologic ultrasound revealed left renal vein Nutcracker syndrome and left renal urine salt crystallization. CT urography showed a few high-density contrast agent shadows in the lower part of the delayed enhanced vaginal cavity and a few high-density sediment shadows in the posterior wall of the bladder. After penicillin for anti-infection treatment, drinking water for diuresis, and amlodipine for blood pressure control, the child's temperature was normal, blood pressure recovered, and urine findings were normal.

One month later, the CSF protein level was 909.0 mg/L, the IgG index was 0.69, the IgG 24-h synthesis rate was 19.82 mg, and serum was negative for anti-sulfatide IgM. ASO was 736 IU/ml, and urine test results were normal. There were no significant changes in peripheral nerve conduction function or lower limb sensory evoked potentials. After 3 months, peripheral nerve conduction function improved slightly. The patient was followed up for approximately 10 months. There was no paraesthesia or other symptoms, and the muscle strength and tension were normal. Peripheral nerve conduction function was improved. Head CT findings returned to normal.

## Discussion and conclusion

Guillain‒Barré syndrome (GBS) is an immune-mediated acute inflammatory peripheral neuropathy. Anti-perineural surface glycolipid antibodies, including anti-sulfatide and anti-ganglioside antibodies, are closely related to the pathogenesis of autoimmune-mediated peripheral neuropathy. Sulfatide is an acidic glycolipid that is abundant in peripheral nerve tissues and mainly distributed in the peripheral nerve myelin sheath. Sulfatide plays an important role in maintaining the structure of the nerve sheath and regulating the transmission of nerve impulses and membrane information [1, 2]. The pathogenic mechanism of anti-sulfatide antibodies in inflammatory peripheral neuropathy is not very clear, which is different from the molecular mimicry hypothesis of anti-ganglioside antibodies, but both are related to immune dysfunction caused by prodromal infection [3]. The patient had a prodromal infection at the beginning of the disease, which was consistent with the disease. The clinical manifestations of anti-sulfatide antibody-associated GBS spectrum disorders are heterogeneous and are related to small fiber neuropathy and painful sensory axon neuropathy, and limb weakness is rare. Electrophysiological examination shows demyelination and/or axonal degeneration, with prolonged distal latency, pain, and paresthesias as typical features [4, 5], all of which were consistent with the findings for this patient. We report the case of a school-age girl who met the diagnosis of anti-sulfatide antibody-associated GBS spectrum disorder with ventricular dilatation, urinary calculi, and autonomic dysfunction, highlighting the differences between this case report and previous reports.

CSF protein-cell separation is an important feature of GBS spectrum diseases. Most of the protein content is normal within a few days after onset, and the protein content in CSF is increased to varying degrees within 2-4 weeks. A total of 30-50% of patients have normal protein content in the first week after onset, and the protein content of 10-30% of patients is still normal in the second week [6]. Therefore, normal CSF protein content cannot rule out GBS. Headache and hydrocephalus are rare complications in patients with GBS, and more than 10 cases have been reported thus far. Hydrocephalus can occur before the onset of GBS symptoms and even recur after the symptoms have improved [7, 8]. However, there has been no report of hydrocephalus secondary to anti-sulfatide antibody-related GBS and no reports of abdominal pain and urinary calculi. At present, the commonly accepted mechanism of hydrocephalus is that increased CSF protein concentration slows reabsorption in the arachnoid granulations. However, the relationship between brain edema and CSF protein levels is not linear. Considering that the patient in this case initially experienced headache and abdominal pain, and head imaging findings suggested that ventricular dilatation and urinary calculi might be possible, we have reason to believe that the autonomic nervous system may play a role in this. Sulfatide antibodies can affect autonomic nerve fibers, and children with abdominal pain experience smooth muscle spasms (which is known because spasmolytic medicines can relieve pain). Furthermore, the paroxysmal flushing of the face, heart rate fluctuations, and sweating that occur in the course of GBS can also be caused by autonomic dysfunction. Headache resulting from neuromodulation may also involve blood vessels rather than just CSF protein increasing due to osmotic pressure changes. Moreover, ventricular dilatation, hydrocephalus, and CSF pressure are not consistent [9]. In this patient, the maximum CSF protein was 3.78 g, and headache was obvious, but the maximum CSF pressure was 170 mmH2O. The disease can be self-limited, and reduced CSF absorption can improve on its own. Most drugs are effective; however, some patients need surgical drainage. The symptoms of this child were relieved after immunotherapy, and no surgical intervention was performed.

GBS spectrum disease is an immune-induced disease. Immunotherapy such as immunoglobulin is effective, but the use of corticosteroids is still controversial. In clinical practice, glucocorticoid therapy is not necessary for patients with mild GBS. However, for some patients with severe GBS, the short-term application of glucocorticoids may reduce the process of inflammation and edema in diseased tissues [10]. In this patient, the treatment effect of two courses of immunoglobulin shock therapy was not satisfactory, and the CSF protein gradually increased, accompanied by severe neuralgia and protein absorption disorder caused by spinal nerve root disease. The symptoms improved after two weeks of low-dose steroid therapy, and no recurrence was observed. Although GBS can be self-limiting, we do not think it is a coincidence that the child's condition did not resolve after 1 month of IVIG treatment but gradually resolved after steroid therapy. The reason for this is that at the beginning, we temporarily administered steroids at only 10 mg qd, and although the child felt some relief from the pain and weakness, the improvement did not last. Therefore, 3 days later, we administered a small maintenance dose of steroids again, which we discontinued after 2 weeks; since then, the symptoms have not recurred. It is suggested that short-term low-dose steroid therapy, especially for anti-sulfatide antibody-associated GBS, should be considered for patients with significantly increased CSF protein with obvious neuralgia and a poor therapeutic effect of immunoglobulin.

## Conclusion

Anti-sulfatide antibody-associated GBS is associated with small fiber peripheral neuropathy, usually manifested as autonomic and peripheral sensory nerve changes, with pain and other sensory abnormalities and autonomic dysfunction as the main manifestations. With the progression of the disease, muscle strength may decrease, which is considered to be related to the deposition of anti-sulfatide antibodies on peripheral nerve axonal terminals, sensory nerves and neurons in the posterior root ganglion. At present, ventricular dilatation and urinary calculi have not been reported in anti-sulfatide antibody-associated GBS. In addition to the dysfunction of nerve root absorption caused by increased CSF protein, dysautonomia may be involved in pain. When the therapeutic effect of immunoglobulin is not satisfactory, a low dose and short course of steroid therapy can be considered. Steroid therapy can be used as a second-line option in patients for whom IVIG treatment is less effective. The recovery of clinical symptoms is faster than that of electrophysiological findings, and the prognosis is good. Definitely, anti-sulfatide antibody and response to steroids has a temporal correlation but causation is difficult to establish via a single case report. Hopefully, more cases will confirm this in the future.

## Data Availability

Not applicable. All data generated or analyzed during this study are included in this published article.

## Abbreviations

GBS: Guillain-Barre Syndrome
CSF: Cerebrospinal fluid
ASO: Anti streptolysin O
OB: Oligoclonal bands
SNAP: Sensory nerve action potential
SCV: Sensory conduction velocity
MRI: Magnetic resonance imaging
ESR: Erythrocyte sedimentation rate
ANA: Antinuclear antibody
